# Sudden suspected anaphylactic shock after multiple tolerated doses of piperacillin-tazobactam: a case report and literature review

**DOI:** 10.3389/fmed.2026.1887086

**Published:** 2026-08-20

**Authors:** Ping Li, Hongli Yu, Xiaomei Mo, Lu Liu, Chang Liu, Zhe Zhang, Meixing Yan

**Affiliations:** 1 Department of Pharmacy, Women and Children’s Hospital, Qingdao University, Qingdao, Shandong, China; 2 No. 971 Hospital of the People’s Liberation Army Navy, Qingdao, Shandong, China

**Keywords:** adverse drug reaction, anaphylactic shock, drug sensitization period, fake antigen theory, piperacillin-tazobactam, toddler

## Abstract

**Background:**

The most well-known adverse reaction of penicillins is anaphylactic shock, which mostly occurs within the first few minutes of the initial administration. However, cases of sudden anaphylactic shock that develop after multiple consecutive doses with no prior adverse events are extremely rare.

**Case presentation:**

This report presents a case of sudden suspected anaphylactic shock in a 20-month-old toddler following several consecutive days of uneventful administration of piperacillin-tazobactam.

**Conclusion:**

This case underscores that the sensitization phase of β-lactam antibiotics may remain occult throughout the entire course of treatment. The absence of severe adverse reactions during early drug administration does not eliminate subsequent hypersensitivity risks, which emphasizes the imperative for clinicians to implement rigorous monitoring during each administration to safeguard pediatric medication safety.

## 1 Introduction

Piperacillin-tazobactam (PIP-TAZ) is a frequently used beta-lactam/beta-lactamase inhibitor combination with excellent tolerability. As this agent exerts a bactericidal effect against Gram-negative pathogens and certain Gram-positive pathogens, it is widely applied in the clinical management of infectious diseases in pediatric patients (1, 2). In clinical practice, the common adverse reactions of PIP-TAZ include gastrointestinal disturbances and cutaneous rashes. Although severe allergic reactions (e.g., anaphylactic shock) have a low incidence rate, they are associated with life-threatening severity (3). Herein we describe a pediatric case of sudden suspected anaphylactic shock occurring after 7 days of continuous uneventful PIP-TAZ intravenous infusion. This study aims to illustrate that patients may develop occult sensitisation risks to β-lactam antibiotics despite uneventful tolerance after multiple administrations in the early phase of a single treatment course, providing evidence-based references for the safe medication management in paediatric clinical practice.

## 2 Case presentation

### 2.1 Pre-hospital outpatient management (19–21 Sep, 2025)

The patient was a 20-month-old Chinese male toddler weighing 11 kg with no chronic underlying diseases and no documented history of drug or food allergy. Initial chief complaint was left cervical lymphadenopathy with persistent fever (peak temperature 38.8 °C). Outpatient laboratory tests showed white blood cell count 15.93 × 10^9^/L, C-reactive protein (CRP) 15.32 mg/L. Cervical ultrasound revealed enlarged lymph nodes (2.4 cm × 1.6 cm) without obvious abscess liquefaction. Empirical intravenous cefotaxime (30 mg/kg every 8 h) was prescribed for 3 consecutive days. After treatment, fever persisted and local lymph node swelling did not regress, indicating insufficient treatment response to third-generation cephalosporin monotherapy.

### 2.2 Inpatient anti-infective therapy (22–29 Sep, 2025)

Upon admission, multiplex real-time PCR was immediately conducted to screen influenza A/B virus, respiratory syncytial virus, adenovirus, and human rhinovirus, and all tests returned negative results. Serological testing for Epstein-Barr virus (EBV) also yielded negative findings. Repeat cervical ultrasound demonstrated enlarged left cervical lymph nodes measuring 2.2 cm × 1.9 cm, indicating a high risk of abscess formation. Serial inflammatory markers showed progressive elevation: white blood cell count 17.41 × 10^9^/L and C-reactive protein (CRP) 62.86 mg/L. Accordingly, a diagnosis of unilateral bacterial cervical lymphadenitis with persistent fever was established. Surgical consultation favored conservative antimicrobial therapy over urgent incision and drainage. Given the suspected production of β-lactamase by the pathogen and treatment failure with the initial cephalosporin regimen, intravenous PIP-TAZ (formulation: piperacillin 2.0 g plus tazobactam 0.25 g) was administered at a dose of 80 mg/kg every 8 h. The 80 mg/kg weight-based dosage mentioned in this manuscript only refers to the piperacillin component; each single infusion delivers a total drug dose of 0.99 g, with a total daily dosage of 2.97 g administered in three separate infusions every 8 h. From September 22 to 29, 2025, the child received a total of 23 scheduled intravenous infusions without observed clinical adverse reactions. Vital signs remained stable throughout each administration, and the patient’s infectious symptoms improved gradually during hospitalization.

### 2.3 Onset of hypersensitivity and emergency resuscitation (night of 29 Sep, 2025)

At 22:30, the 24th infusion of PIP-TAZ was started. Approximately 5 min after infusion initiation, with only 5 mL of solution administered, the child suddenly became inconsolable with severe irritability, accompanied by facial pallor, perioral cyanosis and cold extremities. At symptom onset, blood pressure was immeasurable using a non-invasive cuff sphygmomanometer. Initial vital signs captured at this time point were documented as follows: heart rate of 88 beats/min (a reduction of 30 beats/min relative to the patient’s baseline value), respiratory rate of 35 breaths/min, and peripheral oxygen saturation of 88%. Physical examination revealed prolonged capillary refill time (CRT) > 3 s, cool peripheral extremities, altered mental status, and dry oral mucous membranes. This spectrum of clinical manifestations fulfils the 2024 GA^2^LEN consensus diagnostic criteria for life-threatening systemic anaphylaxis (4).

The Naranjo Adverse Drug Reaction Probability Scale (NAP scale, hereafter referred to as the Naranjo scale) was used to evaluate the causal association between the adverse event and PIP-TAZ, yielding a total score of 5 (detailed scoring results are shown in Table 1), corresponding to a probable adverse drug reaction.

**Table 1:** Evaluation of the patient’s adverse drug reactions via the Naranjo scale.

Questions	Yes	No	Don’t know	Score
1. Are there previous conclusive reports on this reaction?	1	0	0	1
2. Did the adverse event appear after the suspected drug was given?	2	−1	0	2
3. Did the adverse reaction improve when the drug was discontinued or a specific antagonist was given?	1	0	0	1
4. Did the adverse reaction reappear when the drug was readministered?	2	−1	0	0
5. Are there alternative causes that could have caused the reaction?	−1	1	0	0
6. Did the reaction reappear when a placebo was given?	−1	1	0	0
7. Was the drug detected in any body fluid in toxic concentrations?	1	0	0	0
8. Was the reaction more severe when the dose was increased or less severe when the dose was decreased?	1	0	0	0
9. Did the patient have a similar reaction to the same or similar drugs in any previous exposure?	1	0	0	0
10. Was the adverse event confirmed by any objective evidence?	1	0	0	1

Total score: 5. Score classification: definite (≥9), probable (5–8), possible (1–4), doubtful (≤0).

At 22:36, the infusion of PIP-TAZ was terminated immediately. Prompt standardized emergency interventions were administered immediately, including nasal cannula oxygen at 2 L min^−1^, intramuscular epinephrine 0.1 mg (0.01 mg kg^−1^) injected into the anterolateral thigh, intravenous bolus dexamethasone sodium phosphate 3 mg (0.3 mg kg^−1^), and rapid intravenous fluid resuscitation with 0.9% normal saline at 20 mL kg^−1^.

At 22:45, the child gradually regained consciousness with flushed facial skin and relieved perioral cyanosis; blood pressure recovered to 90/60 mmHg and peripheral oxygen saturation reached 99%.

At 22:55, the patient’s vital signs became fully stabilized: heart rate 120 beats/min, respiratory rate 30 breaths/min, blood pressure 111/74 mmHg, peripheral oxygen saturation ranging from 98 to 100%. The extremities were warm, with CRT < 2 s. All abnormal vital signs and clinical manifestations resolved completely within 20 min after rescue, with no subsequent adverse sequelae. The child received continuous vital sign monitoring in the general ward for 24 h after the shock event as post-reaction observation.

### 2.4 Clinical stabilization and discharge (30 Sep–02 Oct, 2025)

No further intravenous interventions were implemented subsequent to the anaphylactic shock episode. Laboratory recheck revealed a white blood cell count of 12.76 × 10^9^ L^−1^, neutrophil absolute count of 6.14 × 10^9^/L, and C-reactive protein (CRP) of 5.45 mg L^−1^. The patient remained clinically stable with no recurrent fever and was discharged on October 2. At discharge, a high-risk allergy alert for PIP-TAZ was added to the electronic medical record. The guardians were counselled to avoid this specific agent pending a comprehensive allergy evaluation. They were also advised that other penicillins, cephalosporins, or other β-lactams should not be empirically avoided but should be considered individually after appropriate skin testing and/or challenge under specialist supervision. Emergency anti-anaphylaxis rescue drugs and equipment must be fully prepared during any subsequent hospital medication exposure.

### 2.5 Patient and parent perspective

The toddler’s parents experienced severe panic and psychological distress during the emergency management of acute anaphylactic shock. The caregivers reported being unaware that life-threatening hypersensitivity reactions might arise following several days of uncomplicated PIP-TAZ administration, and they maintained persistent anxiety regarding the safety of future antibacterial therapy for their child. Clinical pharmacists and pediatricians delivered standardized, streamlined medication-related education to the family. Subsequently, the guardians clearly understood that only PIP-TAZ requires temporary discontinuation. They also acknowledged the clinical importance of undergoing formal assessment by an allergy specialist to identify tolerable alternative β-lactam agents.

## 3 Discussion

β-lactam antibiotics are recognized as one of the primary drug classes associated with drug-induced anaphylactic shock. Approximately 0.7–10% of anaphylactic shock events are attributed to penicillins (5). Due to their rapid onset and severe clinical manifestations, such reactions can be life-threatening if not managed in a timely manner (6). The incidence of type I hypersensitivity to penicillins ranges from approximately 0.01 to 0.05% (7). Traditional type I hypersensitivity theory divides the reaction into two sequential phases: sensitization and effector activation. During primary antigen exposure, B lymphocytes generate specific IgE, which binds to high-affinity FcεRI on mast cells and basophils to establish a sensitized state; upon secondary antigen exposure, antigen-IgE cross-linking triggers mast cell degranulation and release of bioactive mediators, inducing systemic hypersensitivity (8, 9). The immunogenic epitopes driving penicillin allergy arise from covalent conjugation between β-lactam ring hydrolysis metabolites or R1 side-chain haptens and endogenous human serum or tissue proteins, where the side-chain chemical structure serves as the primary allergenic determinant (10).

Clinically, typical β-lactam-induced anaphylaxis generally occurs within minutes after the initial drug administration. Such acute reactions imply that immune sensitization has already been completed covertly prior to the first formal medication exposure, often through unrecognized cross-sensitization to structurally similar drugs or food allergens (11). In sharp contrast, the present case exhibited an extremely atypical clinical pattern: the patient tolerated 23 consecutive PIP-TAZ infusions without observed clinical adverse reactions and developed fulminant anaphylactic shock abruptly during the late treatment course. This paradoxical presentation raised concerns regarding potential non-immune confounding factors or operational errors that might mimic hypersensitivity-like reactions. To exclude medication-related or operational confounders that may mimic acute hypersensitivity reactions, the hospital pharmacy department launched a comprehensive full-chain quality audit immediately after the incident, covering procurement, incoming inspection, storage, dispensing and Pharmacy Intravenous Admixture Services (PIVAS) compounding. Closed-circuit video records documenting the entire compounding procedure were reviewed together with the patient’s family. The identical batch of PIP-TAZ was administered to other inpatients in the same ward and other departments with no adverse events reported. Infusion concentration and rate were strictly implemented in accordance with institutional pediatric standard operating procedures. These systematic investigations did not identify evidence of contamination, preparation error, or excessive infusion rate. All investigation results have been communicated to the patient’s family.

Combining the classic hapten paradigm with the fake antigen theory proposed by Pichler (12) provides one hypothetical explanation for the atypical clinical manifestations observed in our patient, who developed fulminant anaphylactic shock following 23 consecutive well-tolerated infusions. Sustained intravenous exposure to β-lactams facilitates gradual formation of covalent hapten–human serum albumin conjugates (true antigens) over days of repeated dosing. These stable adducts are processed by antigen-presenting cells to stimulate *de novo* synthesis of drug-specific IgE, which immobilizes on mast cell and basophil FcεRI to establish latent sensitization. Concurrently, steady circulating drug concentrations drive antigen-specific mast cell hyporesponsiveness, rendering patients asymptomatic during serial routine infusions. Notably, covalent hapten-protein complexes form slowly over hours under physiological pH, and their low steady-state tissue concentrations fail to reverse this mast cell tolerant phenotype, which explains the complete absence of adverse manifestations throughout the prolonged pre-shock treatment course. During the 24th infusion, however, markedly elevated local drug concentrations rapidly generate abundant non-covalent drug–albumin assemblies termed “fake antigens.” Bearing structural homology to covalent hapten conjugates, fake antigens rapidly cross-link pre-existing drug-specific IgE anchored on mast cell membranes, abrogate established mast cell hyporesponsiveness, and trigger robust degranulation with massive release of inflammatory mediators, culminating in severe anaphylactic shock within minutes of infusion commencement. Other alternative explanations for this clinical phenomenon also exist, including progressive in-treatment sensitization during repeated drug exposure, or prior unrecognized contact with cross-reactive allergen determinants before hospitalization.

From a clinical perspective, atypical anaphylactic shock events consistent with this case have been reported, though they appear uncommon. Babu and Sharmila reported a 7-day-old neonate experiencing near-lethal anaphylaxis on day 6 of continuous cefotaxime therapy following multiple uneventful infusions, with clinical manifestations highly consistent with our pediatric case (13). Moreno-Ancillo et al. further described severe anaphylactic shock in a 4-month-old infant after the third cefotaxime dose (14). Mirroring our diagnostic reasoning, both prior studies confirmed drug-induced anaphylaxis based on pathognomonic clinical features, clear temporal association between drug exposure and symptom onset, and rigorous exclusion of competing differential diagnoses. Collectively, these published case reports illustrate that abrupt, life-threatening hypersensitivity reactions can occasionally arise following several days of uneventful repeated β-lactam exposure. The fake antigen theory further provides supplementary hypothetical mechanistic rationale to interpret this atypical anaphylactic shock phenotype. However, anaphylactic shock can involve both IgE-dependent and non-IgE-mediated pathways, rather than being restricted solely to Immunoglobulin E-mediated type I hypersensitivity. For the present case, this proposed mechanism remains purely speculative, as we were unable to perform assays for drug-specific IgE, basophil activation markers, mast cell activation biomarkers, or drug-protein complexes to verify the aforementioned pathway.

## 4 Limitations of this case report

This case report has two notable limitations. First, serum tryptase assay—the gold-standard biomarker for identifying mast cell activation during acute anaphylaxis—could not be performed, as our institution does not offer this test as a routine laboratory service. Second, skin provocation testing and basophil activation test (BAT) were not conducted because the patient’s guardians declined all drug re-challenge procedures, precluding acquisition of direct laboratory evidence to distinguish IgE-dependent versus non-IgE-mediated immune mechanisms for this reaction. We acknowledge that the Naranjo scale is a general causality assessment tool and cannot confirm the underlying pathogenic mechanism. Accordingly, the anaphylactic shock described in this manuscript constitutes a high-probability clinical suspicion instead of a definitive laboratory-confirmed diagnosis.

## 5 Conclusion

The key takeaway of this case is that hypersensitivity risks associated with β-lactam antibiotics may persist throughout the entire treatment period. No adverse reactions were observed in this pediatric patient after 23 consecutive infusions, yet abrupt shock developed merely 5 min following the 24th administration. This clinical manifestation reasonably indicates that sensitization can remain occult even after repeated drug administration. Nevertheless, this inference lacks direct laboratory evidence from IgE testing and cannot be regarded as a confirmed objective finding. This single case only delivers targeted clinical alerts rather than universal mandatory criteria for intensified full-course monitoring. However, clinicians should reassess the prevailing notion that allergy monitoring is only required upon the initial drug administration. From a practical perspective, medical institutions are recommended to establish a sound medication safety framework covering three core components: full-cycle medication monitoring, permanent availability of emergency drugs and equipment, and standardized emergency response workflows. Multi-dimensional and systematic pharmaceutical interventions can build a robust safety barrier for pediatric patients and guarantee the safety and efficacy of clinical pharmacotherapy. Furthermore, we emphasize that any suspected β-lactam allergy warrants formal consultation with an allergology specialist, instead of empirically avoiding the whole class of β-lactam agents. This strategy helps formulate individualized long-term antibacterial regimens for patients.

## Data Availability

This study is based on a single case report and a literature review; no original datasets were generated or analyzed. Any materials sup porting the findings of this study are available from the corresponding author upon reasonable request at meixing@163.com.
